# Changes in Peak Aerobic Capacity During an 8-Month Period in First-Year Medical Students: A Prospective Pilot Cohort Study

**DOI:** 10.3390/healthcare14183070

**Published:** 2026-09-18

**Authors:** Madeline Harding, Jackson Case, Kelson Knighton, Asis Babun, Benjamin Brooks

**Affiliations:** 1 College of Osteopathic Medicine, Rocky Vista University, Ivins, UT 84738, USA; 2 Department of Clinical Medicine, College of Osteopathic Medicine, Rocky Vista University, Ivins, UT 84738, USA

**Keywords:** VO_2_peak, medical students, cardiorespiratory fitness, metabolic health, indirect calorimetry

## Abstract

**Background/Objectives:** Medical school has long been thought to have a potentially detrimental effect on the health of its students. In this preliminary study, we sought to determine whether students experienced a decline in their cardiorespiratory physical fitness during an 8-month period of their first year of medical school. **Methods:** A single-center, uncontrolled prospective pilot cohort of first-year medical students (n = 13) from a single institution was recruited during their first month of school to undergo VO_2_peak testing via indirect calorimetry. They were re-tested approximately 8 months later near the end of the academic year using the same protocol. Measurements of body mass, body fat percentage, and resting metabolic rate were also taken before VO_2_peak testing. Participants were also asked to complete surveys related to diet, exercise, sleep, and mental health. **Results:** We were unable to recruit our planned sample size of n = 32 based on a priori power analysis. The final sample of 13 participants increased the risk of Type II error and limits the generalizability of these preliminary findings. We observed a statistically significant decrease in mean VO_2_peak from 37.3 mL/kg/min to 35.0 mL/kg/min (mean change = −2.3, 95% CI [−4.3, −0.3], *p* = 0.048; median paired change = −2.1 [IQR: −4.3, −0.6]). An exploratory food-sufficiency measure showed a nominal decrease, whereas other self-reported lifestyle factors, body composition, and mental health measures did not show statistically detectable changes. **Conclusions:** In this small exploratory cohort, VO_2_peak declined over the approximately 8-month observation period. No changes in self-reported lifestyle factors, body composition, or mental health reached preliminary statistical significance.

## 1. Introduction

Medical students face unique challenges, including high academic demands, long study hours, and clinical responsibilities, which can negatively impact both their physical and mental health. While much attention has been given to the mental health of medical students, with high rates of anxiety, depression, and burnout being well documented, the role of metabolic health as a mediator of wellness is underexplored. Cardiovascular fitness (often measured as VO_2_peak) is crucial for overall health and may influence mental well-being and academic performance [1].

The transition from adolescence to university is itself associated with changes in lifestyle and physical activity; medical training may introduce additional academic and behavioral demands that could further affect physical fitness. Existing studies have shown that physical activity can mitigate symptoms of anxiety and depression in a dose-dependent manner, with peak benefits observed between 2 and 7.5 h of exercise per week [2]. However, as medical students progress through their education, physical fitness tends to decline possibly due to increased academic pressures [3,4]. This decline in fitness is often accompanied by body mass gain and worsening mental health outcomes [5]. While these trends are well documented in certain populations, there is limited research on how metabolic health changes over time in medical students and how these changes relate to mental health.

Physical fitness of medical students has potential downstream effects that are not only limited to the health of the students themselves. There is also evidence suggesting a weak but positive correlation between physical fitness and academic performance in medical students. Studies have found that students with higher fitness scores tend to perform better on exams such as the USMLE Step 2 CK [3]. Although academic performance was not assessed in the present study, this association provides additional rationale for monitoring physical fitness during medical training and represents an important area for future research. In addition to the benefit of higher academic achievement, health habits of medical students may one day influence how they counsel patients. Healthcare practitioners who report a healthier lifestyle are more likely to counsel their patients on lifestyle interventions [6,7,8]. Given that medical students will become healthcare professionals responsible for counseling patients on physical activity and other lifestyle interventions, monitoring their own physical fitness and health behaviors may have implications beyond their individual well-being. Understanding and improving the health of medical students offers a wide array of benefits.

The current study was designed to collect detailed, prospective data about a cohort of students during an 8-month period within their first year of medical school. We sought to prospectively measure changes in the VO_2_peak of United States medical students across 8 months of training using indirect calorimetry. Alongside this primary endpoint, we aimed to understand if other critical wellness metrics also deteriorate during this timeframe. The primary, prespecified hypothesis was that VO_2_peak would decline during medical school; all other measures (resting metabolic rate, body composition, diet, physical activity, sleep and mental health) were treated as exploratory secondary outcomes. These measures were selected to provide a more comprehensive assessment of how the demands of medical education may affect health beyond changes in aerobic fitness alone. By establishing exactly what happens to a student’s health during eight months of medical school, this study may help inform students and faculty if preventative interventions should be deployed from “day one” to help students maintain their health throughout their medical careers.

## 2. Methods

### 2.1. Study Design and Participants

This was a prospective longitudinal cohort study designed to evaluate changes in cardiorespiratory fitness and health behaviors through 8 months roughly corresponding to the first year of medical school. The study protocol was reviewed and approved by the Institutional Review Board at the Rocky Vista University (Protocol #2025-106). The study protocol was not registered on any public database. All participants were informed of the study’s purpose, procedures, and potential risks, and all provided written informed consent prior to participation and data collection. The initial study protocol stated that measurements would be taken at three points during the year, but this was later changed to two measurements due to scheduling conflicts. This change occurred before any data analysis was performed. Participants were recruited via word of mouth from the matriculating first-year class at the Utah campus of the Rocky Vista University during the months of July and August 2025. A power calculation (Cohen’s d of 0.5, significance level 0.05, 80% power, single-tailed *t*-test) indicated that we should have recruited 32 students. However, a total of only 13 students volunteered and were enrolled in the study. Since the study did not reach its recruitment target, it should be considered underpowered and exploratory.

Prior to participation, students completed a basic medical screening survey administered via Qualtrics (Provo, UT, USA). Inclusion criteria were matriculating first-year students in the Utah campus of the Rocky Vista University School of Osteopathic Medicine campus cohort, age ≥18 years, and willingness/ability to complete a maximal cycle-ergometer exercise test. Exclusion criteria were self-reported medical comorbidities (e.g., uncontrolled cardiovascular or pulmonary disease) or musculoskeletal injuries that would preclude maximal exercise testing. Screening responses were reviewed by Jackson Case and Madeline Harding to determine clearance for maximal exercise testing. No participants were excluded at screening and no adverse events occurred during exercise testing.

### 2.2. Procedures

Data collection for the initial timepoint occurred during August 2025, approximately one month after students began their first year of medical school. Data collection for the second timepoint occurred at the end of the month of March 2026, approximately 2 months before the end of their first year. The study therefore evaluates change across an 8-month interval within the first academic year. At each session, participants underwent physiological testing and completed several questionnaires on their mobile devices. Participants were instructed to abstain from caffeine for at least 4 h before testing; however, time of day, hydration status, and physical activity immediately preceding testing were not formally standardized.

The primary endpoint of this study was cardiorespiratory fitness, quantified by VO_2_peak. VO_2_peak was measured via indirect calorimetry using the VO_2_ Master device, a validated wearable metabolic analyzer (VO_2_ Master Health Sensors Inc., Vernon, BC, Canada) [9,10]. The analyzer measures minute ventilation, tidal volume, respiratory frequency, oxygen uptake, and expired oxygen fraction; it does not measure expired carbon dioxide, and therefore respiratory exchange ratio could not be derived. The machine was calibrated by the manufacturer within 1 year and was calibrated according to manufacturer recommendations prior to testing using ambient-air gas calibration and flow calibration with a 3 L syringe. No internal coefficient of variation or intraclass correlation coefficient was calculated for repeated VO_2_peak measurements in the present cohort. The reported 4–6% coefficient of variation is based on the previously published literature and was not independently validated in the present cohort. Expired-gas data were processed as a 30 s rolling average, and VO_2_peak was defined as the highest 30 s averaged VO_2_ attained during the test. Participants breathed through a face mask fitted to ensure an airtight seal.

Participants underwent an incremental exercise protocol on a stationary cycle ergometer, JOROTO X2PRO (Li’ang Health Science and Technology Co., Ltd., Beijing, China), with resistance increasing progressively until exhaustion. The protocol began with a 4 min warm-up at a workload below 50 watts, followed by 1 min at 50 watts. Resistance was then increased by 50 watts every minute until the participant reached maximal effort. Participants followed an individual cadence that was comfortable for them. All participants followed the same testing protocol regardless of estimated fitness. The protocol concluded with a 3 min cool-down at a workload below 50 watts. A stepped/ramp cycle protocol with incremental loading was selected because incremental cycle-ergometer testing is a validated method for eliciting peak aerobic capacity, and shorter incremental phases (as short as ~7 min) can still yield valid peak VO_2_ values in adults. This protocol was adapted from a previously validated method for assessing VO_2_peak [11,12].

Several other datapoints were collected as secondary endpoints. Resting metabolic rate (RMR) was measured using an FDA approved MedGem indirect calorimeter (Microlife Medical Home Solutions, Inc., Golden, CO, USA) [13]. Participants were instructed to abstain from consuming caffeine or any calories for at least 4 h prior to testing. Body mass and body fat percentage were determined using the Tanita BC-533 bioelectrical impedance scale (Tanita Corporation, Tokyo, Japan) which has been used in previous longitudinal body composition studies [14]. For use of the bioelectrical impedance scale, hydration status, recent exercise, food intake, bladder status, and time of day were not controlled for or standardized. The same equipment was used at both timepoints. Dietary habits were evaluated using the Rapid Eating Assessment for Participants-Short Version (REAP-S v.2) which has been validated specifically in 1st-year medical students [15]. Total metabolic equivalents (METs) were calculated using the well-known International Physical Activity Questionnaire Short Form (IPAQ-SF) [16]. Physical activity data were processed and scored in accordance with the official International Physical Activity Questionnaire (IPAQ) guidelines. Total weekly metabolic equivalent (MET) minutes were calculated using standard weighting factors: 8.0 METs for vigorous activity, 4.0 METs for moderate activity, and 3.3 METs for walking. Upon reviewing the data for extreme values, no reported activity category exceeded the IPAQ truncation threshold of 16 h per day, so no adjustments were necessary. Furthermore, all participant surveys were fully completed, requiring no statistical handling for missing data. Sleep quality was assessed using a 1–10 scale according to the Single-Item Sleep Quality Scale (SQS) [17]. Depression and anxiety were screened using the Patient Health Questionnaire-9 (PHQ-9) and the Generalized Anxiety Disorder-7 (GAD-7) scale [18,19].

### 2.3. Statistical Analysis

Data for metabolic and anthropomorphic measurements were recorded in a Microsoft Excel spreadsheet (Microsoft 365 version, Microsoft Corporation, Redmond, WA, USA). Data collected from Qualtrics surveys were downloaded as Excel files and combined with the metabolic and anthropomorphic measures. The data were then anonymized and analyzed. Data were analyzed using Python 3.14.3 (Python Software Foundation, Wilmington, DE, USA) with the libraries pandas (v2,2), SciPy (v1.17.1), and matplotlib (v3.9).

The study was initially powered a priori for 32 participants; however, only 13 participants were ultimately enrolled. This substantially reduced the study’s statistical power and increased the risk of a Type II error (β), in which a true difference or association exists but is not detected as statistically significant. Therefore, the study’s negative or nonsignificant findings should be interpreted cautiously, as the limited sample size may have resulted in insufficient power to detect an effect that was actually present. These findings are best interpreted as those of a pilot study, providing preliminary evidence regarding the feasibility of the protocol and an initial estimate of the effect size for future, adequately powered studies. Given the small sample size, non-parametric tests were used for inferential statistics. Start-of-year and end-of-year differences were analyzed using the Wilcoxon signed-rank test. The Wilcoxon signed-rank test was computed using SciPy v1.17.1 (Python). The exact permutation distribution was used for all comparisons. Ties in absolute differences were resolved by midranking prior to computation of the exact test statistic. Effect sizes are calculated using Cohen’s dz. Mean values are reported along with the 95% confidence intervals [95% CI] for all values. For statistical analysis, only the beginning and ending values for VO_2_peak were considered to be our primary endpoint. All secondary comparisons were considered exploratory and hypothesis-generating. No adjustment for multiple comparisons was performed; therefore, secondary *p*-values should be interpreted as nominal and not confirmatory.

## 3. Results

Baseline characteristics are reported in Table 1. A total of 13 first-year medical students (six male, seven female) enrolled in and completed the study. At baseline, the mean age of the cohort was 24.5 [23.2, 25.9] years. Initial VO_2_peak for the cohort was 37.3 [32.8, 42.3] mL/kg/min. Baseline mental health screening indicated minimal depression and anxiety, with average PHQ-9 and GAD-7 scores of 3.9 [2.5, 5.4] and 3.0 [1.2, 5.4], respectively.

**Figure 1:**
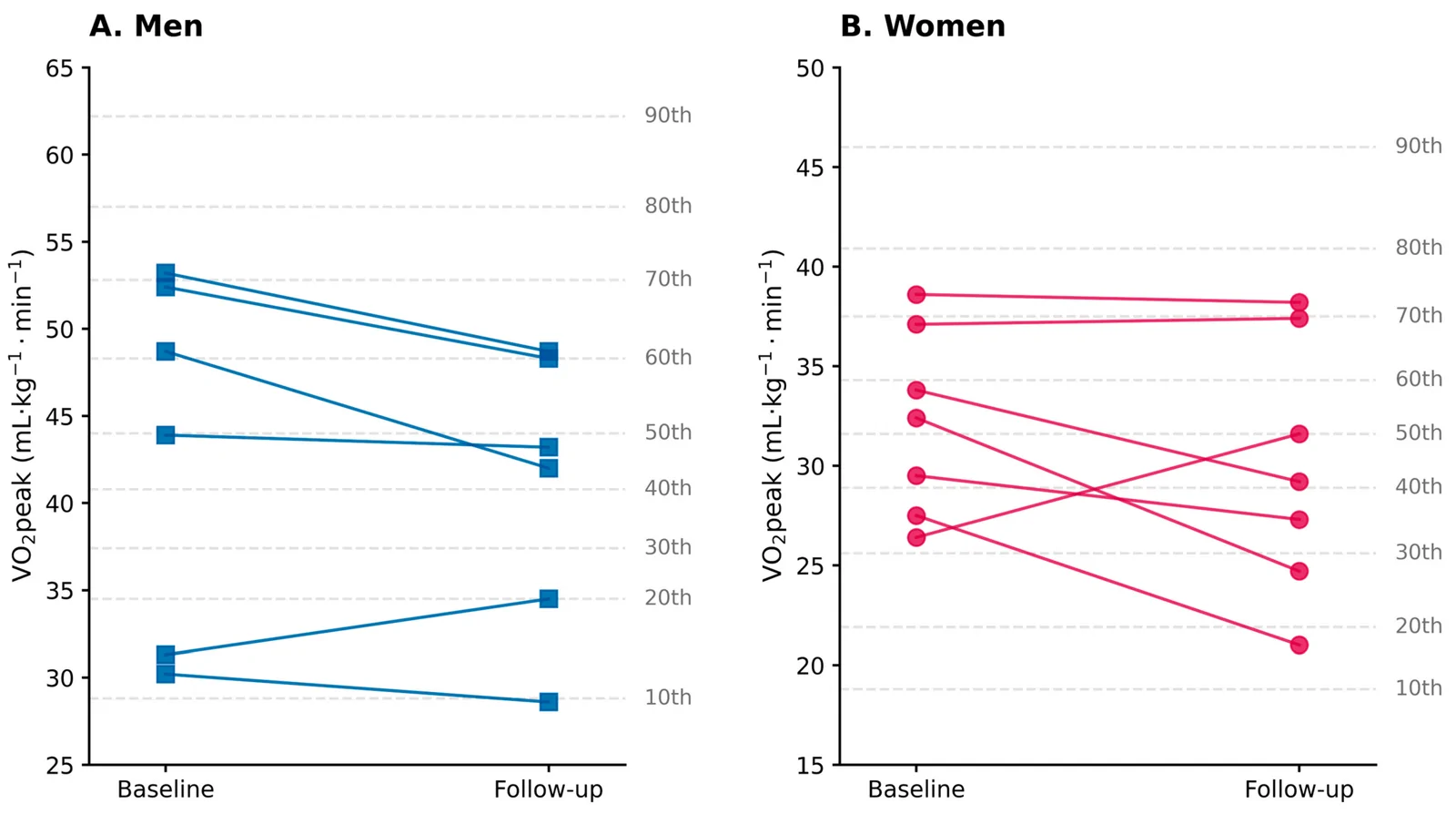
Individual changes in cardiorespiratory fitness (VO_2_peak, mL·kg^−1^·min^−1^) from baseline to follow-up, stratified by sex: (**A**) men and (**B**) women. Each line represents a single participant. Horizontal dashed lines indicate age- and sex-specific normative percentiles (10th–90th). Benchmarked against the sex- and age-specific FRIEND cycle-ergometry reference standards for adults aged 20–29 years [20].

Over the approximately 8-month observation period within the first academic year, the cohort of 13 medical students exhibited a statistically significant decrease in cardiorespiratory fitness, with VO_2_peak dropping from 37.3 [30.2, 43.9] to 35.0 [28.6, 42.0] mL/kg/min (a reduction of 2.3 [−4.3, −0.3]; *p* = 0.048, d = −0.603). The median paired change was −2.1 mL/kg/min [IQR: −4.3, −0.6], consistent with a downward shift across participants. Increases in total body mass and body fat percentage were observed but were not statistically significant. Self-reported physical activity levels remained stable. Total weekly MET expenditure showed a negligible increase of 13 (*p* = 1.000), and average weekday sitting time decreased non-significantly by 0.9 h (*p* = 0.297). While overall dietary scores were largely unchanged, there was a nominally significant exploratory decline in the REAP-S food sufficiency subscale from 9.7 [8.3, 10.8] to 8.2 [7.0, 8.5] (*p* = 0.041, d = −0.658). The food sufficiency subscale incorporates items related to meal skipping, adequate protein intake, and consumption of foods rich in calcium and vitamin D. Psychological well-being and sleep metrics remained stable. Shifts in depressive symptoms (PHQ-9), anxiety (GAD-7), and sleep quality (Sleep SQS) were minimal and not statistically significant. Data for all measures taken at baseline and at follow-up are found in Table 2. Individual-level, sex-stratified trajectory plots (as in Figure 1) were not generated for every secondary outcome in Table 2 because of the very small per-sex subgroup sizes (n = 6 men, n = 7 women), which risk being over-interpreted as reliable sex differences.

The majority, though not all students, saw a decrease in their VO_2_peak from baseline to follow-up (Figure 1). In all, 10/13 students had at least a small decrease in their measured VO_2_peak values over the approximately 8-month observation period.

Overall, VO_2_peak decreased in most participants, with greater variability observed among women.

## 4. Discussion

This exploratory pilot study was designed to prospectively characterize change in cardiorespiratory fitness during an 8-month period within the first year of medical school; findings may be of interest to medical educators and student wellness programs. We observed a decrease in cardiorespiratory fitness over 8 months in this small cohort of American medical students. Compared to the FRIEND registry reference standards for cycle ergometry in adults aged 20 to 29 years, the cohort demonstrated average baseline cardiorespiratory fitness, with both men (43.3 mL/kg/min) and women (32.2 mL/kg/min) ranking near the 50th percentile. Following the measured decline, the mean VO_2peak_ for both sexes fell to approximately the 40th percentile (40.9 mL/kg/min for men and 29.9 mL/kg/min for women) [20]. The observed mean decline of approximately 6% is notable, although its magnitude approaches reported test–retest variability for exercise testing and should therefore be interpreted cautiously. For generally healthy people in their 20s, VO_2_peak is expected to decline about 3–6% over the course of a decade [21,22]. The observation of this change over several months supports further investigation of whether cardiorespiratory fitness changes during the early period of medical training.

The clinical significance of the observed 2.3 mL/kg/min decrease in this young, generally healthy cohort is uncertain. For context, a change in 1 mL/kg/min is associated with altered risk for hypertension, dyslipidemia, and metabolic syndrome [22]. However, the exact clinical significance of this finding in our study population is unclear. Our study population was generally younger and healthier than many of the populations where small changes in aerobic capacity are most related to clinically significant outcomes. Brehm et al. published one of the few studies that have looked at medical student health longitudinally. They found that between the first and fourth year of school, American medical students had decreased cardiovascular fitness (as measured by the Forestry step test) and small changes in certain metabolic biomarkers [23]. Our study was not able to measure any blood biomarkers associated with decreased VO_2_peak. Possible mechanisms underlying the observed decline include reduced incidental physical activity, increased sedentary academic time, psychological stress associated with the transition to medical training, disrupted sleep and dietary routines, and loss of organized team/sport participation; our uncontrolled design cannot directly test these mechanisms. We also do not know if the change we observed was only transient or if it represents a loss that will not be recovered [24].

To our knowledge, this is the first study to demonstrate a decline in cardiovascular fitness in medical students using VO_2_peak via indirect calorimetry. Decreases in other metrics of cardiovascular fitness such as a 1.5 mile timed run [4] or a step test [23] have been reported before, but none measure the function of the cardiopulmonary system with the same precision as a VO_2_peak test using calibrated equipment. Another strength of our research is the prospective design as much of the data on medical student wellness relies on cross-sectional surveys and retrospective analysis. While a prior retrospective study at our institution found early declines in student mental health and exercise [25], our prospective cohort did not mirror these effects. Finally, our study had 100% retention which eliminates attrition bias. However, other forms of bias such as selection bias are very likely present.

The exploratory secondary outcomes did not reveal a consistent parallel deterioration across measured behavioral or wellness domains. Body mass and body fat increased modestly, while resting metabolic rate, self-reported physical activity, mental health, and sleep measures did not show statistically detectable changes. Overall dietary quality also remained relatively stable, although the food sufficiency subscale showed a nominally significant exploratory decrease. This discordance may indicate that the observed change in VO_2_peak was not accompanied by detectable changes in self-reported physical activity, mental health, sleep, or overall dietary quality; however, the study was not powered to establish absence of change, and measurement limitations, particularly reliance on self-report, preclude strong interpretation. The decline in food sufficiency should therefore be considered hypothesis-generating and warrants further investigation in larger studies.

This study also has several significant limitations. Foremost among the limitations is the small sample size of only 13 participants. Our initial power calculations predicted that we would need to recruit many more participants, but we observed a larger effect size than anticipated. Our cohort was also subject to a number of biases. Students who volunteer for a rigorous VO_2_peak study are likely more health-conscious and physically active than the general medical student population at baseline. Another limitation is that desirability bias may have skewed our secondary self-reported questionnaires, particularly regarding mental health and exercise volume, potentially masking steeper behavioral declines. Total daily energy expenditure, energy intake, and changes in academic workload were not objectively measured or controlled and represent additional unmeasured potential confounders. Additionally, this study did not include a comparison group, baseline testing occurred after matriculation rather than before it, the observation period did not span the complete academic year, the study was underpowered relative to its a priori calculation, multiple secondary comparisons were not corrected for multiplicity, and regression to the mean, seasonal variation, and measurement error could not be excluded as contributors to the observed change. Self-report instruments (IPAQ-SF, REAP-S, PHQ-9, GAD-7, SQS) are also susceptible to recall and social-desirability bias, and bioelectrical impedance estimates of body composition are sensitive to hydration and testing conditions that were not fully standardized. The 4 h caffeine-abstinence period may not have been sufficient to fully eliminate caffeine’s effects on exercise performance, given its variable half-life and potential ergogenic effects. Additionally, because the portable analyzer did not measure expired carbon dioxide, RER could not be calculated, and neither a VO_2_ plateau nor a verification bout was documented; we therefore report VO_2_peak rather than VO_2_max. In addition, the 50 W·min^−1^ ramp produced relatively short incremental phases in the least-fit participants, which may have contributed to underestimation of true maximal aerobic capacity. Although the same device and protocol were used at both assessments, measurement variability cannot be excluded as a contributor to the observed within-participant change.

One participant repeated testing more than 48 h after an equipment-related data-capture failure; only the technically valid repeat measurement was analyzed. Prior research demonstrates good reproducibility of cycle-ergometer VO_2_peak measurements across repeated tests performed within one to two weeks [11,26]. Accordingly, the repeat test was retained for analysis. Some amount of variance is expected without true changes in aerobic capacity because of causes such as regression to the mean, seasonal variation, and protocol or device inconsistency. Finally, this is a single-center study, and the results may not perfectly generalize to different institutions. More specifically, these findings reflect 13 volunteers from a single institution and should not be generalized to American medical students as a whole.

Our limited sample size also greatly limited our ability to correlate changes in VO_2_peak with other changes in student health. Future studies may be designed with sufficient statistical power to understand how VO_2_peak in medical students interacts with mental health, diet, and exercise. Future studies should include objective measures, such as accelerometry, to complement self-reported assessments and reduce recall and reporting bias. Future research would also benefit from larger, more diverse cohorts to increase the generatability of the results. The field of medical student wellness may also benefit from more prospective data. This could include trials on which interventions are most effective for increasing student physical and mental well-being. To strengthen these prospective designs, researchers should incorporate pre-matriculation baseline testing with repeated assessments across the full academic year, utilize matched comparison groups across multicenter cohorts, and ensure standardized device calibration and test preparation. Furthermore, future trials should employ rigorous analysis plans that clearly distinguish confirmatory from exploratory outcomes.

## 5. Conclusions

In this small, single-institution cohort of 13 first-year medical students in the United States, VO_2_peak declined over an approximately 8-month period within the first academic year. An exploratory dietary food-sufficiency measure also declined, whereas other secondary outcomes did not show statistically detectable changes. Given the small, self-selected sample, absence of a comparison group, incomplete academic-year observation, and potential measurement variability, these findings should be considered preliminary. Larger multicenter studies beginning before matriculation are needed to determine whether the observed change is reproducible, identify contributing factors, and evaluate whether early wellness interventions can preserve cardiorespiratory fitness during medical training.

## Figures and Tables

**Table 1:** Participant characteristics stratified by sex. Values are presented as mean [95% CI] for the total sample (n = 13), males (n = 6), and females (n = 7). Demographic variables include age and height. Biometrics include body mass, body fat percentage, peak oxygen consumption (VO_2_peak), and maximal heart rate (HR). Mental health and wellness measures include Patient Health Questionnaire-9 (PHQ-9), Generalized Anxiety Disorder-7 (GAD-7), and Sleep Quality Scale (SQS). Physical activity was assessed using the International Physical Activity Questionnaire Short Form (IPAQ-SF), with metabolic equivalent (MET) minutes per week calculated for vigorous, moderate, and walking activity, as well as total METs and average weekday sitting time. Dietary quality was assessed using the Rapid Eating Assessment for Participants–Shortened Version (REAP-S v.2), with subscale and total scores reported (higher scores indicate healthier dietary patterns). Gender-stratified values are descriptive given the small subgroup sizes (n = 6, n = 7) and are not intended to support inferential gender comparisons.

Characteristic	Total (n = 13)	Male (n = 6)	Female (n = 7)
Demographics			
Age (years)	24.5 [23.2, 25.9]	26.2 [23.8, 28.2]	23.2 [22.4, 23.7]
Height (cm)	171.4 [164.7, 178.1]	182.4 [179.3, 189.1]	162.0 [160.6, 164.0]
Biometrics			
Weight (kg)	73.0 [65.9, 81.4]	82.6 [71.2, 94.7]	64.8 [60.5, 68.7]
Body fat (%)	26.4 [22.2, 30.1]	21.3 [16.0, 26.3]	30.9 [27.7, 33.7]
VO_2_peak (mL/kg/min)	37.3 [32.8, 42.3]	43.3 [36.1, 50.0]	32.2 [29.1, 35.4]
Max HR (bpm)	184.7 [181.3, 188.2]	186.7 [181.0, 192.3]	183.0 [179.1, 186.4]
Resting metabolic rate (kcal/day)	1919 [1683, 2207]	2315 [1997, 2695]	1579 [1464, 1686]
Mental health & wellness			
PHQ-9 score	3.9 [2.5, 5.4]	4.3 [2.5, 6.2]	3.6 [1.6, 5.7]
GAD-7 score	3.0 [1.2, 5.4]	4.3 [1.0, 9.0]	1.9 [0.6, 3.3]
Sleep quality (SQS, 0–10)	6.7 [6.2, 7.2]	6.8 [6.3, 7.3]	6.6 [5.9, 7.4]
Physical activity (IPAQ-SF)			
Vigorous METs/wk [8.0 × days × min]	954 [538, 1415]	987 [240, 1827]	926 [514, 1371]
Moderate METs/wk [4.0 × days × min]	438 [243, 648]	333 [113, 560]	529 [229, 840]
Walking METs/wk [3.3 × days × min]	506 [248, 855]	635 [223, 1235]	396 [156, 745]
Total METs/wk	1899 [1304, 2529]	1955 [1064, 3079]	1850 [1068, 2542]
Sitting time (h/weekday)	8.6 [7.2, 10.0]	9.5 [7.2, 11.8]	7.8 [6.5, 9.1]
Dietary quality—REAP-S v.2 (0–3 scale; higher = healthier)			
Food sufficiency subscale (max = 12)	9.7 [8.3, 10.8]	8.7 [6.2, 10.5]	10.6 [9.4, 11.6]
Healthy eating pattern subscale (max = 21)	9.6 [7.8, 11.3]	8.3 [6.2, 10.7]	10.7 [8.3, 12.9]
Low-nutrient-density foods subscale (max = 24)	15.8 [14.4, 17.2]	13.8 [12.5, 15.2]	17.4 [16.1, 18.9]
Exercise subscale (max = 3)	2.5 [2.2, 2.8]	2.5 [1.8, 3.0]	2.6 [2.1, 2.9]
Total REAP-S score (max = 60)	37.6 [35.1, 40.2]	33.3 [31.7, 35.0]	41.3 [39.4, 43.1]

**Table 2:** Changes in outcomes from baseline (Start) to follow-up (End). Values are mean [95% CI]; Delta represents mean change (End − Start). *p*-Values from Wilcoxon signed-rank tests; effect sizes reported as Cohen’s dz. The primary outcome is cardiorespiratory fitness as defined by VO_2_peak. Secondary outcomes include body composition, mental health (PHQ-9, GAD-7), sleep (SQS), physical activity (IPAQ-SF; MET-min/week and sitting time), and dietary quality (REAP-S v.2; higher scores indicate healthier diet). *p* < 0.05 denoted by *. For the primary outcome (VO_2_peak), the median paired change and IQR are reported alongside the mean change and CI given the small sample size. Bold values denote the primary outcome measure.

Outcome	Start	End	Delta	*p*-Value	dz
Cardiorespiratory fitness					
**VO_2_peak (mL/kg/min)**	**37.3 [32.8, 42.3]**	**35.0 [30.6, 39.5]**	**−2.3 [−4.2, −0.3]; median −2.1 [IQR: −4.3, −0.6]**	0.048 *	**−0.603**
Body composition					
Weight (kg)	73.0 [65.8, 81.6]	74.5 [67.0, 83.3]	+1.5 [−0.2, +3.5]	0.216	0.423
Body fat (%)	26.4 [22.3, 30.2]	27.0 [22.8, 31.0]	+0.6 [−0.6, +1.7]	0.312	0.264
Resting metabolic rate (kcal/day)	1918 [1671, 2222]	1943 [1697, 2208]	+25 [−128, +183]	0.8	0.084
Mental health					
PHQ-9	3.9 [2.5, 5.4]	4.2 [2.6, 5.8]	+0.2 [−1.8, +2.2]	0.748	0.062
GAD-7	3.0 [1.2, 5.4]	2.8 [1.5, 4.1]	−0.2 [−3.0, +2.0]	0.879	−0.048
Sleep					
Sleep SQS (0–10)	6.7 [6.2, 7.2]	6.1 [5.4, 6.8]	−0.6 [−1.5, +0.2]	0.236	−0.382
Physical activity (IPAQ-SF)					
Vigorous METs/wk	954 [538, 1415]	932 [646, 1274]	−22 [−511, +434]	0.851	0.027
Moderate METs/wk	438 [243, 648]	308 [169, 474]	−131 [−414, +172]	0.331	−0.233
Walking METs/wk	506 [248, 855]	672 [372, 998]	+165 [−218, +569]	0.413	0.218
Total METs/wk	1899 [1304, 2529]	1912 [1333, 2580]	+13 [−733, +801]	1	0.04
Sitting time (h/weekday)	8.6 [7.2, 10.0]	7.7 [6.2, 9.2]	−0.9 [−2.2, +0.3]	0.297	−0.352
Dietary quality—REAP-S v.2 (0–3 scale; higher = healthier)					
Food sufficiency subscale (max = 12)	9.7 [8.3, 10.8]	8.2 [7.0, 9.5]	−1.5 [−2.5, −0.2]	0.041 *	−0.658
Healthy eating pattern subscale (max = 21)	9.6 [7.8, 11.3]	9.6 [8.5, 10.8]	+0.0 [−1.8, +1.8]	0.906	0
Low-nutrient-density foods subscale (max = 24)	15.8 [14.4, 17.2]	16.4 [14.3, 18.3]	+0.6 [−0.7, +1.9]	0.453	0.249
Exercise subscale (max = 3)	2.5 [2.2, 2.8]	2.2 [1.8, 2.6]	−0.3 [−0.8, +0.2]	0.406	−0.325
Total REAP-S score (max = 60)	37.6 [35.1, 40.2]	36.5 [32.7, 39.8]	−1.2 [−4.6, +2.2]	0.734	−0.177

## Data Availability

The data presented in this study are available on request from the corresponding author due to participant confidentiality reasons.
